# The Etiology and Epidemiology of Pediatric Facial Fractures in North-Western Romania: A 10-Year Retrospective Study

**DOI:** 10.3390/children9070932

**Published:** 2022-06-21

**Authors:** Paul Andrei Țenț, Raluca Iulia Juncar, Abel Emanuel Moca, Rahela Tabita Moca, Mihai Juncar

**Affiliations:** 1 Department of Dentistry, Faculty of Medicine and Pharmacy, University of Oradea, 10 Piața 1 Decembrie Street, 410073 Oradea, Romania; tent_andrei@yahoo.com (P.A.Ț.); ralucajuncar@yahoo.ro (R.I.J.); mihaijuncar@gmail.com (M.J.); 2 Doctoral School of Biomedical Sciences, University of Oradea, 1 Universității Street, 410087 Oradea, Romania; rahelamoca@gmail.com

**Keywords:** pediatric facial fractures, etiology, epidemiology, Romania

## Abstract

Pediatric facial fractures are not as common as facial fractures occurring in the adult population. Their therapeutic approach is different because they affect patients with active growth, and have an etiology and epidemiology that vary depending on different cultural, religious and demographic factors. This research aimed to identify the main factors involved in the etiology of pediatric facial fractures, as well as the epidemiology of pediatric facial fractures in a sample of children and adolescents from North-Western Romania. This 10-year retrospective study was performed in a tertiary center for oral and maxillofacial surgery in North-Western Romania. Medical files of patients that were admitted between 1 January 2002 and 31 December 2022 were analyzed. Pediatric patients aged 0 to 18 years were included in this study. The final sample consisted of 142 children and adolescents diagnosed with facial fractures, with this number representing 14.1% of all patients affected by facial fractures. Most frequently, fractures were identified in the 13–18 age group (78.9%, *n* = 112), which were more often associated with fractures caused by interpersonal violence than caused by road traffic accidents, falls or animal attacks. Boys were more affected (88%, *n* = 125), and were more frequently associated with fractures caused by interpersonal violence. The most frequently identified etiological factors included interpersonal violence (50%, *n* = 71), falls (18.3%, *n* = 26) and road traffic accidents (11.3%, *n* = 16). In terms of location, the mandible was the most affected facial bone structure (66.2%, *n* = 94), and patients with mandibular fractures were more frequently associated with fractures caused by interpersonal violence. The incidence of pediatric facial fractures should be lowered because they may interfere with the proper development of the facial skeleton. Establishing measures aimed at preventing interpersonal violence, as well as other causes involved in the etiology of facial fractures is imperative.

## 1. Introduction

Facial trauma can profoundly affect a victim’s social life, and have a negative impact on a person’s overall quality of life [1]. Facial trauma occurring in adults is the most common diagnosed and treated pathology in maxillofacial surgery services [2], and involves complex treatment, which often requires interdisciplinary collaboration in order to minimize any long-term negative effects [3].

Compared to facial fractures occurring in adult patients, pediatric facial fractures are not as common [4], but their therapeutic approach represents a serious challenge for physicians, especially due to the fact that they occur in actively growing patients [5]. Generally, pediatric facial fractures occurring in children under the age of 5 years do not exceed an incidence rate of 5% [4], but they are more frequent in adolescent patients [5].

Pediatric facial fractures have an etiology that often varies, mostly depending on the age of the child, but the most common causes include fall injuries [6,7], sports accidents [8], motor vehicle accidents [9], or even child abuse [10]. However, the etiology can vary significantly depending on the cultural, religious and demographic background of the patient as well [11]. Age, the presence of intramaxillary teeth, or the topography of lesions influences the treatment of pediatric facial fractures and the choice of a specific therapeutic approach [5,12]. Facial fractures can occur in the frontal bone, the orbita, the nasal bone, the zygomatic bone, the maxilla or the mandible [12]. In the pediatric population, mandibular fractures are among the most common facial fractures, reaching an incidence of up to 50% of all pediatric facial fractures [13]. Their treatment varies from close observation, associated with soft diet and analgesics, to intermaxillary fixation used for short periods of time [13].

Pediatric facial fractures can cause skeletal deformities due to fracture-induced growth defects [13], and should be rapidly and correctly diagnosed and managed, even if the lesions tend to be less severe [14]. Globally, the incidence and etiology of facial fractures vary according to each population studied [2], and this trend is maintained in the pediatric population as well. In the geographic region investigated in this research, the authors did not identify any studies that presented the etiology and epidemiology of pediatric facial fractures. Knowing these aspects is important for preventing and optimally managing facial fractures in a pediatric population [15].

The aim of this research was to identify the main factors involved in the etiology of pediatric facial fractures, as well as the epidemiology of pediatric facial fractures in a sample of children and adolescents from North-Western Romania.

## 2. Materials and Methods

### 2.1. Ethical Considerations

The study was approved by the Ethics Committee of the University of Oradea (IRB No. 3402/15.04.2018), and was conducted in accordance with the standards described by the 2008 Declaration of Helsinki and its later amendments. All 18-year-old patients signed an informed consent form at the time of their admission in the medical institution regarding the anonymous use of their medical data for future scientific studies. For minor patients (under the age of 18), the informed consent forms for the anonymous use of patients’ medical data were signed by their parents or legal guardians.

### 2.2. Participants and Data Collection

This ten-year retrospective study was conducted in a tertiary center for oral and maxillofacial surgery from North-Western Romania. All medical files belonging to patients hospitalized in this institution between 1 January 2002 and 31 December 2011 were initially analyzed.

The following inclusion criteria were applied: patients up to 18 years of age, who had at least one facial fracture line at the time of admission, and an acute traumatic episode in the history of the disease; patients for whom the clinical diagnosis was confirmed by a paraclinical examination method (radiographic examination or computed tomographic examination); and patients whose traumatic fractures were treated in the host hospital.

The exclusion criteria were as follows: patients aged 19 years or older, with no facial skeletal fractures, or with pathological bone fractures; and patients for whom the clinical diagnosis was not confirmed by any additional paraclinical examination, who were treated in another hospital, or with incomplete information in the medical records.

The data needed to conduct this research were extracted from patients’ medical records. The following variables were analyzed: gender of patients (boys, girls), age of patients (0–6 years, 7–12 years, 13–18 years), living environment of patients (urban, rural), etiology of trauma, location of fracture lines in the facial skeleton (mandible, midface, combined) and the presence of associated lesions in the soft tissues (hematomas, lacerations, abrasions).

All observation sheets were double-checked by the author responsible for data collection and by a member of the team responsible for compiling the statistics. This was performed in order to avoid bias.

### 2.3. Statistical Analysis

The statistical analysis was performed using IBM SPSS Statistics 26 (IBM, Chicago, IL, USA) and Microsoft Office Excel and Word 2013 (Microsoft, Redmond, WA, USA). All categorical variables were expressed as an absolute or in a percentage form, and were tested using Fisher’s Exact Test. Z-tests with Bonferroni corrections were performed in order to detail the results after obtaining the contingency tables.

## 3. Results

**Figure 1:**
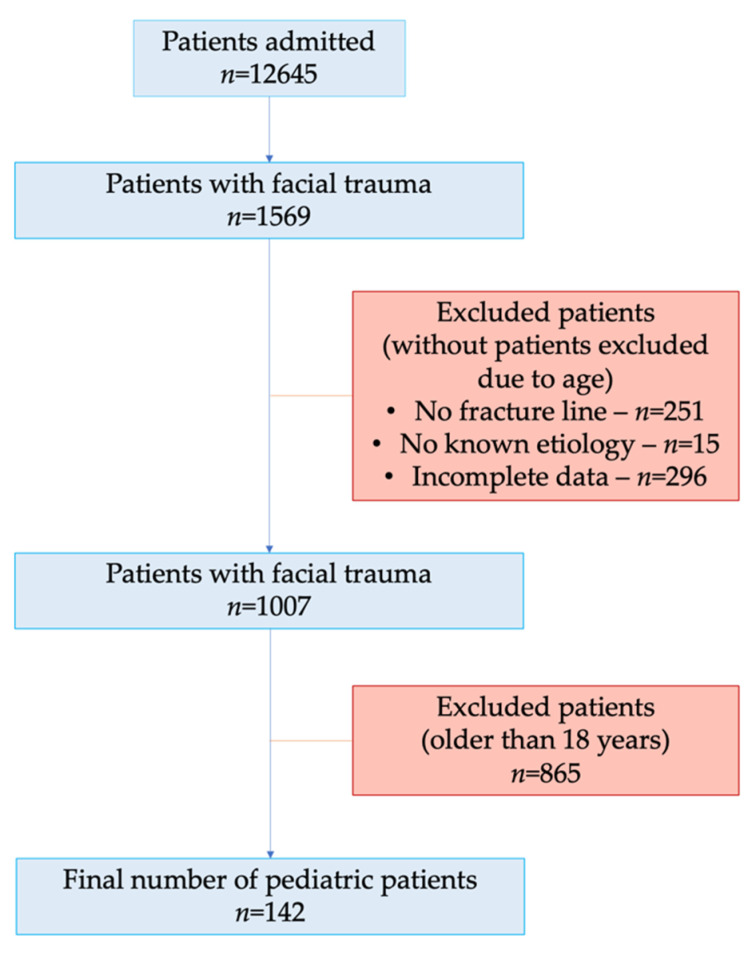
Study flowchart.

During the 10-year timespan analyzed, a total of 12,645 patients were hospitalized, of which 1569 had facial trauma. After applying the exclusion criteria, 1427 patients were excluded, and 142 patients were included in this study (Figure 1). The age-based exclusion criterion was applied as a final exclusion criteria in order to calculate the total percentage of pediatric patients affected by facial fractures out of the total number of patients (children, adolescents, adults) affected by facial fractures.

The data in Table 1 show the demographic characteristics of the patients investigated. Of the total sample of patients with facial fractures that were admitted during the 10-year timespan, before applying the age-based exclusion criteria (1007 patients), 14.1% (*n* = 142) of the fractures occurred in pediatric patients (with ages between 0 and 18 years). The majority of pediatric patients with fractures were boys (88%, *n* = 125), from urban areas (61.3%, *n* = 87), the most common etiology being interpersonal violence (50%, *n* = 71), followed by fall trauma (18.3%, *n* = 26), and road traffic accidents (11.3%, *n* = 16). Regarding the associated soft tissue lesions, 43.7% (*n* = 62) of children had hematomas, 28.2% (*n* = 40) had lacerations, and 33.1% (*n* = 47) had abrasions. Regarding the location of fractures, 66.2% (*n* = 94) had strictly mandibular fractures, 25.3% (*n* = 36) had only midface fractures, and 8.5% (*n* = 12) had combined fractures (affecting both the midface and the mandible).

**Figure 2:**
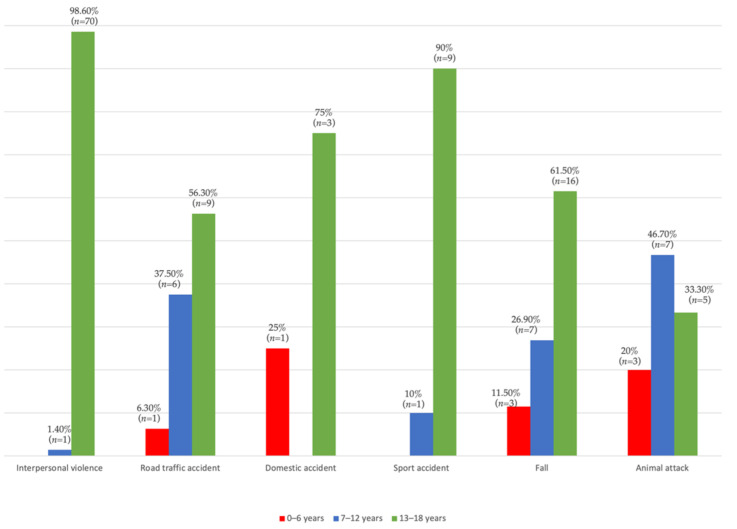
Distribution according to age and etiology.

**Figure 3:**
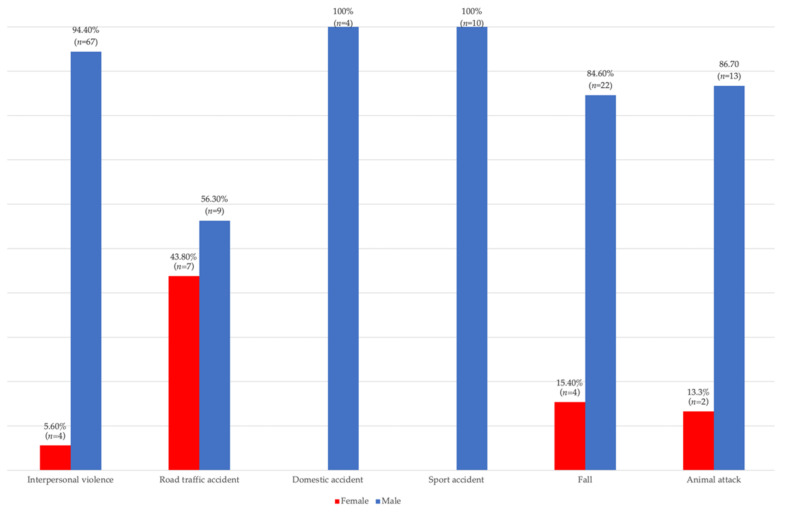
Distribution according to gender and etiology.

**Figure 4:**
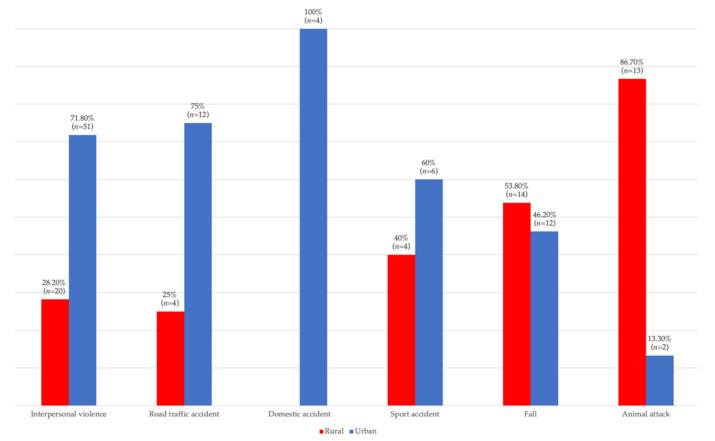
Distribution according to living environment and etiology.

Data in Figure 2, Figure 3 and Figure 4 show the distribution of pediatric patients according to the age, gender, living environment and etiology of trauma. Differences between groups were statistically significant according to Fisher’s Exact Test, and Z tests with a Bonferroni correction showed that patients aged 0–6 years were more frequently associated with fractures caused by domestic accidents or animal attacks than caused by interpersonal violence, and patients aged 7–12 years were more frequently associated with fractures caused by road traffic accidents, falls or animal attacks than those caused by interpersonal violence. However, patients aged 13–18 years were more frequently associated with fractures caused by interpersonal violence than those caused by road traffic accidents, falls or animal attacks (*p* < 0.001). Regarding the gender of the patients, it was observed that girls were associated more frequently with fractures caused by road traffic accidents than those caused by interpersonal violence, and boys were more frequently associated with fractures caused by interpersonal violence than fractures caused by road traffic accidents (*p* = 0.004). Regarding the living environment of the patients, it was found that children and adolescents from rural areas were more frequently associated with fractures caused by animal attacks than fractures caused by interpersonal violence, road traffic accidents or domestic accidents, and children and adolescents from urban areas were more frequently associated with fractures caused by interpersonal violence, road traffic accidents or domestic accidents than those caused by animal attacks (*p* < 0.001).

**Figure 5:**
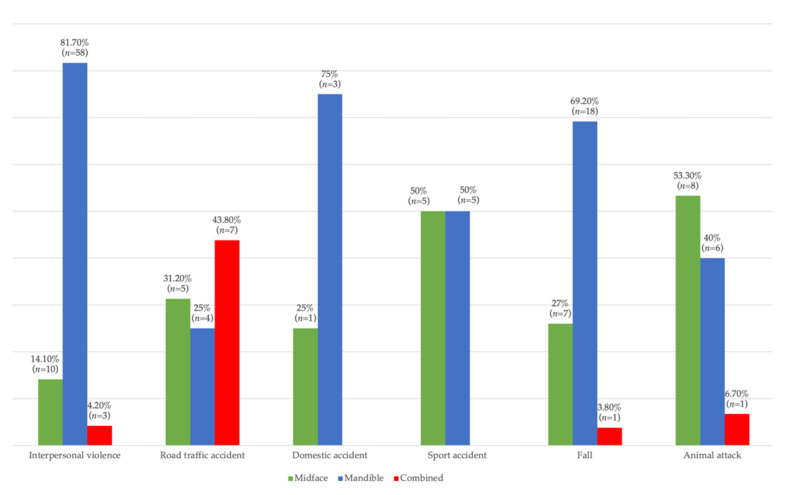
Distribution according to line of fracture and etiology.

The distribution of pediatric patients related to the location of the fracture line and the etiology of trauma is shown in Figure 5. Differences between groups were statistically significant according to Fisher’s Exact Test (*p* < 0.001), and Z tests with a Bonferroni correction showed that children and adolescents with midface fractures were associated more frequently with fractures caused by animal attacks than caused by interpersonal violence, and patients with mandibular fractures were more frequently associated with fractures caused by interpersonal violence than fractures caused by animal attacks. Children and adolescents with combined fractures were associated more frequently with fractures caused by road traffic accidents than those caused by interpersonal violence or falls.

Regarding the soft tissues associated lesions, it was observed that children and adolescents without hematomas were more frequently associated with fractures caused by interpersonal violence than fractures caused by road traffic accidents, falls or animal attacks, and patients with hematomas were more frequently associated with fractures caused by road traffic accidents, falls or animal attacks than fractures caused by interpersonal violence (*p* < 0.001). Patients that did not have any lacerations were more frequently associated with fractures caused by interpersonal violence than fractures caused by road traffic accidents, falls or animal attacks, and children with lacerations were more frequently associated with road traffic accidents, falls, animal attacks than those caused by interpersonal violence (*p* < 0.001). Children and adolescents without abrasions were more frequently associated with fractures caused by interpersonal violence than by road traffic accidents, falls or animal attacks, and children with abrasions were more frequently associated with fractures caused by road traffic accidents, falls or animal attacks than those caused by interpersonal violence (*p* < 0.001). The data are detailed in Table 2.

## 4. Discussion

Pediatric facial fractures had different etiologies, and their incidence varied according to patients’ age, gender and living environment. They are most common in the 13–18 years age group, with interpersonal violence playing a major role in their occurrence. Pediatric facial fractures, although of great importance and with major potential negative effects in the short and long term, do not have a well-characterized epidemiology [16]. They are generally rare, and account for less than 15% of the total number of facial fractures [17]. However, a significant part of the morbidity in the pediatric population is caused by craniofacial fractures [18]. The treatment of facial fractures in children and adolescents differs from the treatment of fractures in adults, considering the fact that pediatric patients have active growth and development [19]. Knowing the etiology and epidemiology of pediatric facial fractures in a specific population is essential for ideal therapeutic management, as well as for successful prevention.

In this research, pediatric facial fractures accounted for 14.1% of all fractures diagnosed for patients admitted in the host institution. The results are consistent with other studies, which reported an incidence of 14.7% [4], 14.6% [20] or 14.1% [21], all of which were below the 15% threshold. A single study was identified that reported an incidence of only 1.01% of facial fractures in the pediatric population under the age of 16 years in India [22], well below the incidence reported in the present study.

Patients were distributed based on age, gender and living environment. The pediatric patients were distributed in three distinct age groups, respectively 0–6 years, 7–12 years and 13–18 years. This distribution was preferred because it comprises three important periods in the development of pediatric patients, namely preschool, school and adolescence [23], but also because it was used in other similar studies [20,23]. The incidence of pediatric facial fractures was influenced by these investigated variables. Thus, the incidence in children under the age of 6 years was 5.6%, a result identical to that obtained by Vyas et al. [4]. Other studies reported a higher incidence in the preschool population [20]. The consensus is, however, that in general, the total number of pediatric patients diagnosed with facial fractures increases with age [4,24].

Regarding the influence of patients’ gender on the incidence of pediatric facial fractures, it was observed that the most affected were boys, who, in our study, accounted for 88% (*n* = 125) of the total number of patients investigated. In general, the literature reports a higher incidence of pediatric fractures in male patients [4,25], which is consistent with our results. In school-age and adolescent patients, the explanation could be correlated with the increased interest in sports, but also with the changing nature of adolescents. In adolescence, the tendency to make risky choices and to have exaggerated emotionally reactions is high [26], thus making adolescent patients prone to various accidents [27]. Studies that report an equally distributed incidence of facial fractures among boys and girls were also identified [28].

The etiology of pediatric facial fractures differs depending on the populations investigated, and involves social, cultural and environmental factors that cause differences between countries [20]. In younger children, most fractures are caused by falls or the practice of various sports [29,30], while in adolescents interpersonal violence is one of the dominant etiological factors responsible for the occurrence of facial fractures [31]. In this study, the main etiological factor in the occurrence of facial fractures was interpersonal violence, which is responsible for 50% of the total number of pediatric facial fractures identified. Additionally, it is important to highlight the fact that out of the total number of cases with fractures caused by interpersonal violence, 98.6% (*n* = 70) occurred in adolescent patients (13–18 years old). Other etiological factors identified were falls, road traffic accidents, animal attacks, sports accidents and domestic accidents.

Pediatric facial fractures most commonly concern the mandible [16,32,33]. This is due to the prominent position of the mandible in the facial skeleton [34]. In the study conducted on the pediatric population from North-Western Romania, the mandibular fractures were the most frequent facial fractures as well, constituting 66.2% (*n* = 94) of the total number of fractures diagnosed. Mandibular fractures were the most common fractures caused by interpersonal violence, domestic accidents and falls. Midface fractures are not as common in children and adolescent as mandibular fractures. This is mainly due to the fact that the facial skeleton and paranasal sinuses of children are not fully developed, leading to craniofacial disproportions [35]. In our study, as well, the midface fractures had a much lower incidence than the mandibular fractures, but they were predominant among fractures caused by animal attacks. Combined fractures were the most common facial fractures that occurred in road traffic accidents. This is due to the high kinetic energy developed in accidents [36].

Complications that occur as a result of facial fractures may also affect soft tissues. In this study, we identified hematomas, lacerations and abrasions caused by facial fractures. In contrast to the results of our study where hematomas were the main associated soft tissue lesions (43.7%), Ferreira et al. reported lacerations as the most common soft tissue-associated lesion [36].

It is the authors‘ belief that this study provides important information regarding the etiology and epidemiology of pediatric facial fractures in a population from North-Western Romania. This information can be used to implement pediatric facial fractures prevention measures, but also to better prepare physicians and nurses for the therapeutic management of facial fractures that occur in children and adolescents.

However, there were some limitations identified in this research. Although the medical institution in which this retrospective study was performed is an oral and maxillofacial surgery hospital, it probably mainly treats the population from North-Western Romania, as it is located in this part of the country; therefore, the etiology and epidemiology of pediatric facial fractures may be different in other regions of the country. At the same time, the study is retrospective, so that data taken from patients’ medical records may be incorrectly or incompletely provided at the time of examination and admission of patients. The exact etiology of the trauma could be misreported. Given that the study population was pediatric, the suspicion of domestic abuse must be raised.

## 5. Conclusions

The incidence of pediatric facial fractures was 14.1%, most of which occurred in patients aged 13–18 years. Boys were the most affected by facial fractures, and the incidence in urban areas was higher than in rural areas. Regarding the causes that led to pediatric facial fractures, it was found that personal violence was responsible for causing half of all pediatric facial fractures. The mandible was the most affected facial bone structure, and the most common soft tissue injuries were hematomas. Overall, all types of fractures require a lower incidence, as they are unwanted events, and in pediatric patients they may interfere with the proper development of the facial skeleton. Measures aimed at preventing interpersonal violence, as well as other causes involved in the etiology of facial fractures are imperative.

## Figures and Tables

**Table 1:** Distribution of the patients according to the various variables.

Variable	Value
Age (*n*, %)	
>18 years	865 (85.9%)
≤18 years	142 (14.1%)
**Age groups (*n*, %)**	
0–6 years	8 (5.6%) (0.8% from all facial fractures)
7–12 years	22 (15.5%) (2.2% from all facial fractures)
13–18 years	112 (78.9%) (11.1% from all facial fractures)
**Gender (*n*, %)**	
Girls	17 (12%)
Boys	125 (88%)
**Living environment (*n*, %)**	
Rural	55 (38.7%)
Urban	87 (61.3%)
**Etiology (*n*, %)**	
Interpersonal violence	71 (50%)
Fall	26 (18.3%)
Road traffic accident	16 (11.3%)
Animal attack	15 (10.6%)
Sport accident	10 (7%)
Domestic accident	4 (2.8%)
**Fracture line location (*n*, %)**	
Mandibular fractures	94 (66.2%)
Midface fractures	36 (25.3%)
Combined fractures	12 (8.5%)
**Soft tissue associated lesions (*n*, %)**	
Hematomas	62 (43.7%)
Lacerations	40 (28.2%)
Abrasions	47 (33.1%)

*n*, number; %, percentage.

**Table 2:** Distribution of the pediatric patients according to the presence of soft tissue associated lesions and etiology of trauma.

Etiology		Interpersonal Violence	Road Traffic Accident	Domestic Accident	Sport Accident	Fall	Animal Attack	*p* *
Hematomas								
No	*n*	56	3	2	4	12	3	<0.001
	%	78.9%	18.8%	50%	40%	46.2%	20%	
Yes	*n*	15	13	2	6	14	12	
	%	21.1%	81.3%	50%	60%	53.8%	80%	
**Lacerations**								
No	*n*	64	7	2	10	13	6	<0.001
	%	90.1%	43.8%	50%	100%	50%	40%	
Yes	*n*	7	9	2	0	13	9	
	%	9.9%	56.3%	50%	0%	50%	60%	
**Abrasions**								
No	*n*	61	3	2	10	13	6	<0.001
	%	85.9%	18.8%	50%	100%	50%	40%	
Yes	*n*	10	13	2	0	13	9	
	%	14.1%	81.3%	50%	0%	50%	60%	

*n*, number; %, percentage; * Fisher’s Exact Test.

## Data Availability

The data presented in this study are available on request from the corresponding authors. The data are not publicly available due to privacy reasons.
